# Human-Centric Lighting Research and Policy in the Melanopsin Age

**DOI:** 10.1177/23727322231196896

**Published:** 2023-10-26

**Authors:** Manuel Spitschan, Daniel S. Joyce

**Affiliations:** 1 TUM School of Medicine & Health, Technical University of Munich, Munich, Germany; 2 TUM Institute for Advanced Study (TUM-IAS), Technical University of Munich, Garching, Germany; 3 Max Planck Institute for Biological Cybernetics, Max Planck Research Group Translational Sensory & Circadian Neuroscience, Tübingen, Germany; 4 Centre for Health Research, University of Southern Queensland, Ipswich, Queensland, Australia; 5 School of Psychology and Wellbeing, University of Southern Queensland, Ipswich, Queensland, Australia; 6 Department of Psychology, University of Nevada, Reno, Reno, Nevada, USA

**Keywords:** light, melanopsin, ipRGCs, non–image-forming effects of light, light equity

## Abstract

Beyond visual function, specialized light-sensitive retinal circuits involving the photopigment melanopsin drive critical aspects of human physiology and behavior, including sleep–wake rhythms, hormone production, mood, and cognition. Fundamental discoveries of visual neurobiology dating back to the 1990s have given rise to strong interest from the lighting industry in optimizing lighting to benefit health. Consequently, evidence-based recommendations, regulations, and policies need to translate current knowledge of neurobiology into practice. Here, reviewing recent advances in understanding of NIF circuits in humans leads to proposed strategies to optimize electric lighting. Highlighted knowledge gaps must be addressed urgently, as well as the challenge of developing personalized, adaptive NIF lighting interventions accounting for complex individual differences in physiology, behavior, and environment. Finally, lighting equity issues appear in the context of marginalized groups, who have traditionally been underserved in research on both fundamental visual processes and applied lighting. Biologically optimal light is a fundamental environmental right.

## Introduction

The last 30 years have discovered photoreceptor classes in the human retina that drive physiology and behavior; this has revolutionized how people measure, craft, and use light. The discovery of these intrinsically photosensitive retinal ganglion cells (ipRGCs)—alongside the maturation of human circadian science and chronobiology as scientific fields—has raised the central question of how light and lighting can optimally support human health and wellbeing. Overviewing the current knowledge on this topic highlights gaps, suggesting a roadmap for research and policy that enhances humans’ unique relationship with light.

### Current State of Knowledge

#### Canonical Rod- and Cone-Based Photoreception

Until the late 1990s, the known photoreceptors in the human eye were the *rods* and the *cones*. The rods subserve vision in dim light, allowing for the rudimentary perception of shapes and forms without much spatial detail. The cones, on the other hand, operate under moderate to bright light conditions and underlie our vision and visual perception of form, spatial detail, motion, and color though sensing the long (L cone), medium (M cone), and short (S cone) wavelengths of visible light. Both rod and cone function has been extensively studied in animal models and humans for almost two centuries, using a wide range of neurophysiological, psychophysiological, and psychophysical methods. In-depth studies led to the discovery of the postreceptoral channels, which combine the signals from the cones to produce pathways encoding luminance (underpinning our perception of brightness), red–green and blue–yellow color information.

#### Novel Melanopsin Photoreception and ipRGC Function

The photopigment *melanopsin* was first discovered in the skin cells of frogs, which change their pigmentation based on the light–dark conditions of the environment (Provencio et al., 1998). Importantly, melanopsin is also expressed in a subset of retinal ganglion cells (RGCs) to transduce light information from the eye to the brain. These RGCs, named ipRGCs, encode light information without input from the rods and cones, as melanopsin is directly expressed in the cell bodies and processes (Berson et al., 2002; Dacey et al., 2005; Do, 2019; Hattar et al., 2002; Lucas et al., 2003; Provencio et al., 1998; Rodgers et al., 2018b; Rollag et al., 2003; Schmidt et al., 2011; Spitschan, 2019).

Functional differences distinguish rods, cones, and the ipRGCs: While rods and cones encode changes in contrast—discarding the information of overall ambient illumination—the ipRGCs encode the intensity of illumination, thereby uniquely encoding overall environmental light level (Dacey et al., 2005). Interactions with the rods and cones enable ipRGCs to signal a very wide range of light levels (Lucas et al., 2012).

## Effects of Light on Circadian, Neuroendocrine, and Sleep Physiology

The human pineal gland produces a hormone called melatonin. Melatonin, sometimes also wrongly called the “sleep hormone,” is not produced during the day and rises in production around 1 to 3 hours before habitual bedtime, continuing to be secreted throughout the night. In 1980, Lewy et al. (1980) discovered that the production of melatonin at night is suppressed by exposure to very bright light. Independent of the discovery of melanopsin around the same time, the spectral sensitivity of melatonin suppression was characterized for the first time in the early 2000s. At the time, the action spectrum was found to be distinct from the spectral sensitivity of the rods and cones, pointing to evidence for another class of photoreceptor (Brainard et al., 2001; Thapan et al., 2001). Later work summarizing the available scientific evidence on melatonin suppression clearly linked melanopsin and melatonin suppression (Brown, 2020; Giménez et al., 2022; Nowozin et al., 2017; Prayag et al., 2019), with a possible but inconclusive role for the S cones (Brown et al., 2021; Spitschan, Lazar, et al., 2019; St Hilaire et al., 2022).

Melatonin suppression is an acute effect of light, occurring over the course of 30 minutes or less. Exposure to light also has a much more fundamental influence on our physiology, by acting as a zeitgeber, an environmental “time giver” to synchronize the circadian clock and shift it (Khalsa et al., 2003; Minors et al., 1991; Rüger et al., 2013). Exposure to light in the morning advances the circadian clock, and exposure to light at night delays the circadian clock, a phenomenon described by the phase–response curve.

Further to circadian phase shifting, light can influence alertness, sleep, and cardiovascular, thermoregulatory, and metabolic function. Melatonin suppression and circadian phase shifting are separate functions: Some stimuli can delay circadian phase without modifying melatonin secretion. To what extent the nonvisual effects of light correspond to a unitary system remains the subject of active research.

### Mechanistic Evidence on the NIF Effects of Light

The retinohypothalamic pathway underlying the so-called non–image-forming (NIF) effects of light connects the retina with the suprachiasmatic nucleus (SCN) in the hypothalamus that is the primary circadian oscillator. Most of the evidence on NIF effects of light at present is descriptive, in the sense that studies typically examine the response to parametric changes in light stimuli, differing in intensity (leading to a dose–response curve), wavelength (leading to an action spectrum), duration (leading to a duration–response curve), and time of exposure (leading to a phase–response curve) (Zeitzer & Lok, 2022).

#### Field and Translational Evidence for NIF Effects

Most studies characterizing NIF effects use carefully and well-controlled stimuli under laboratory conditions. This methodology allows the removal of confounding factors. As a consequence, while these studies are informative of the neurobiological pathways underlying the NIF effects, they are of limited use in the real world. A growing set of evidence has examined the impact of light and light exposure under lifelike, real-world conditions. These studies, enabled by the availability of small, wearable light loggers (Hartmeyer & Andersen, 2023; Hartmeyer et al., 2022; Spitschan et al., 2022), have established some key links between real-world light exposure and a range of outcomes, such as sleep architecture (Wams et al., 2017), circadian entrainment (Woelders et al., 2017), and body mass index (BMI) (Reid et al., 2014).

#### Recent Developments in Metrology

The discovery of the melanopsin cells has led to novel developments in metrology for optical radiation—the science of measurement of light. Metrology is a very mature discipline. For light, several well-established metrics exist for quantifying the brightness and color of illumination to the human observer. As these functions are subserved by the cones (brightness under photopic, daylight-level conditions and color) and the rods (brightness under scotopic, dim conditions), they are unsuitable for quantifying the effect of light on melanopsin/ipRGCs and associated nonvisual functions. There have been several proposals for incorporating this novel dimension of photoreception (Lucas et al., 2014). Built upon previous efforts to develop a consensus, the International Commission on Illumination (Commission Internationale d’Eclairage; CIE) published a novel International Standard, CIE S 026/E:2018 in 2018, containing a set of standard spectral sensitivities (CIE, 2018). CIE S026 remains the standard for the physiologically relevant quantification of light and lighting and is available in a range of convenient software tools, including the open-access platform luox (Spitschan et al., 2021).

Beyond the quantities to be used for quantifying NIF effects, several other recent technological developments support the accurate characterisation of NIF effects. This includes miniaturized wearable light loggers, allowing for the measurement of light exposure under naturalistic conditions, and novel technology to capture multispectral images in a retinally relevant fashion.

### Knowledge Gaps

The previous section highlighted the current state of knowledge. This section identifies knowledge gaps that require further research.

#### Integration of Rod–Cone Signals With ipRGCs and Novel Photoreceptor-Based Metrology

It is well-established that ipRGCs, which are intrinsically photosensitive through melanopsin, also receive cone and rod input from their synapses. This means that ipRGCs respond to light directly (through melanopsin) and indirectly (through rod–cone inputs). In some ipRGCs examined in the primate retina, the S cone input is inhibitory—suppressing ipRGC activity—while the rod, L, and M cone input is excitatory (Dacey et al., 2005). The inhibitory S cone input at the neurophysiological level has been confirmed through in vivo studies in humans, demonstrating that the pupillary reflex is driven by an opponent S cone input: Activation of the S cones leads to a dilation of the pupil (compared to expected constriction of the pupil) (Spitschan et al., 2014; Woelders et al., 2018). As a consequence, the cones and rods modulate retinal input for NIF function at the earliest stage.

To what extent the cones and rods are involved in other NIF functions, including melatonin suppression, circadian phase shifting, and alertness, is not clear. How exactly the rods and cones contribute to NIF vision, with what weights and under which conditions, must become the focus of targeted research to result in more nuanced photoreceptor integration models.

#### Integration of Light With Other Signals in Circadian, Neuroendocrine, and Sleep Physiology

Light is considered the primary driver for NIF function. However, it is well known from animal studies that signals unrelated to light, so-called nonphotic zeitgebers, can impact the circadian system. In humans, the effects of exercise (Youngstedt et al., 2019) and meals (Wehrens et al., 2017) on the circadian clock have been evaluated in an isolated fashion, while under real-world conditions, these co-occur with behavior and light exposure (Coirolo et al., 2022; Youngstedt et al., 2022). It is not clear to what extent these timing signals can compete and facilitate circadian entrainment.

#### Individual Differences in Light Exposure and Light Equity

Under real-world, lifelike conditions, light exposure is highly variable, not only for a given individual but also between individuals differing in health status, geographical location, socioeconomic status, and occupation (Campbell et al., 1988; Crowley et al., 2015; Dumont & Beaulieu, 2007; Okudaira et al., 1983; Reid et al., 2014; Savides et al., 1986; Smolders et al., 2013). While some targeted studies have generated pair-wise or factor-wise comparisons in light exposure between different groups, a generalist framework for measuring and predicting light exposure across demographic groups currently does not exist. A key question surrounding light exposure is that of light equity: Who has access to physiologically sound light? Who is exposed to light at the wrong time?

##### Individual Differences in Light Sensitivity

According to over-whelming evidence, individuals differ in their NIF response to light (Chellappa, 2021; Spitschan & Santhi, 2022). One recent study examining the dose–response curve for melatonin suppression found that the most sensitive individual in their study was about 30× more sensitive than the least sensitive individual (Phillips et al., 2019). A range of factors can explain this difference (Swope et al., 2023), but a comprehensive model is currently not available. This may include both state, such as pupil size (Eto et al., 2021; Gaddy et al., 1993; Higuchi et al., 2008) or caffeine (Wright et al., 1997a, 1997b), and trait factors, including lens transmittance and genetics. While it is known that there are genetic polymorphisms of the melanopsin gene OPN4 (Higuchi et al., 2013; Rodgers et al., 2018a, 2018b; Roecklein et al., 2009), as well as genes controlling circadian and sleep processes (Burns et al., 2023; Chang et al., 2019), the extent to which individual variability can be attributed to genetics is not clear.

However, for sample sizes that may yield evidence for the genetic underpinnings, there are no convenient assays for NIF function that can be deployed at scale. Ways forward could lie in a variety of different techniques, including miniaturized wearable light sensors (Casson, 2023; Hartmeyer & Andersen, 2023; Hartmeyer et al., 2022; Joyce et al., 2020; Mohamed et al., 2021; Spitschan et al., 2022; Stampfli et al., 2023); smartphone-deployed apps probing alertness, cognition, and other parameters (Gardesevic et al., 2022; Klein et al., 2021; Shatte & Teague, 2020); ambulatory systems for measuring hormone concentrations continuously (Grant et al., 2022); and novel nonintrusive ways of measuring sleep–wake rhythms over long periods (Alkalih et al., 2022).

#### Mapping the Parametric Space of Light to Physiological Responses

Light can vary in a range of parameters. Some of these have been characterized extensively, including intensity, wavelength, duration, and timing of exposure. Of course, under natural conditions, light exposure is much more complex and complicated to capture. Even under fixed illumination conditions, the eyes move, thereby displacing the retinal image multiple times a second (Spitschan, 2021). The spatial configuration of natural scenes is also highly complex, though statistically regular. To what extent these parameters of light can influence NIF function is not clear, not least as the parameter space is too high-dimensional for simple explorations. As an example, consider the simple experiment of examining how duration and intensity of illumination interact in driving NIF responses. To characterize the intensity–response behavior, one would typically need at least four different illumination intensities in order to be able to construct a four-parameter dose–response function. For duration, the same holds. Consequently, the entire space of all possible combinations of intensity and durations contains 16 distinct combinations. This is impossible to capture in a within-subjects protocol and, in a between-subjects design, requires a reasonable sample size per cell. Given that a typical circadian phase shifting experiment takes some 32 hours or longer, the combinatorial explosion is prohibitive vis-à-vis the cost of experiments.

Recently, a series of studies demonstrated that sequences of short flashes at the millisecond scale delivered over the course of 1 hour can lead to stronger circadian phase shifts than 1-hour exposure of continuous light at the same illuminance (Joyce et al., 2022a, 2022b; Najjar & Zeitzer, 2016). These “circadian illusions” suggest that fewer photons can sometimes lead to a stronger physiological response, offering a novel method for maximizing circadian responses to light while minimizing energy use. These responses likely arise from complex temporal integration of rod, cone, and ipRGC signals. While duration and intensity properties have been addressed before (Joyce et al., 2022a, 2022b), there are still many combinations of parameters to be explored.

### Roadmap for Research and Policy

Considering the knowns and unknowns of lighting in relation to NIF vision, consider the following roadmap.

1. Address knowledge gaps through adversarial collaborations and international consortia.

At present, scientific consensuses for many effects of NIF lighting are yet to be reached (Moore-Ede et al., 2023), and most scientific efforts elucidating the NIF effects of light are run by single laboratories or small-group collaborations of a handful of laboratories. Given the combinatorically explosive nature of mapping out NIF effects across various light-related parameters, such an approach is unlikely to yield the evidence needed to capture NIF effects under various parametric stimuli, which of course is necessary for the development of accurate models of NIF function. One alternative is to build an international consortium, which would coordinate a comprehensive, but not competitive, scientific campaign to yield high-quality data. Integrated into such a program could be mechanisms of reproducibility and replication to generate robust evidence for making policy. One mechanism to arbitrate between different models of NIF photoreception is adversarial collaborations (Rakow, 2022): Two or more groups of opposing views, or supportive of different models, work on the same research question.

2. Cumulative data gathering and sharing: allowing the development of accurate models that aid implementation.

A key success in understanding NIF effects has been the aggregation of data from disjoint data sets and different laboratories. Some of these efforts have largely used informal techniques of accessing the data, including “data thieving,” i.e., the reconstruction of data from graphical summaries (Burda et al., 2017; Drevon et al., 2017; Marin et al., 2017). Of course, in any data reconstruction effort, there is loss in information. A better way would be to adopt a stance that data sharing becomes normalized and not the exception. Several initiatives enable such a sharing, most notably the National Sleep Research Resource (Zhang et al., 2018). Making data findable, accessible, interoperable, and reusable (FAIR) (Wilkinson et al., 2016) must be a key priority. The development of common measures for data collection (Farber et al., 2023), common data models, metadata descriptors, reporting standards for light interventions (Knoop et al., 2019; Spitschan et al., 2023; Spitschan, Stefani, et al., 2019; Veitch & Knoop, 2020), and analysis pipelines (Hammad et al., 2023; Spitschan et al., 2021) must underpin large-scale data integration efforts. This requires dedicated effort, such as the ongoing professionalization of research software engineering and its integration as a core tool in contemporary scientific research (Horsfall et al., 2023).

3. Moving away from WEIRD samples to incorporate diversity.

Many studies on the NIF effects of light have been completed in Europe or the United States. Consequently, the knowledge base may not represent people in general. Aspects of this phenomenon are widely known in psychology and behavioral scenes as Western, educated, industrialized, rich, and democratic (WEIRD) (Henrich et al., 2010b, 2010a), which encapsulates most psychological phenomena. Whether or not there are key genetic differences in light sensitivity due to genetic ancestry (Smith et al., 2009) remains an open question, and it is therefore important to create a diverse and inclusive evidence base that incorporates non-WEIRD data. Relatedly, it will be critical to consider differences in light exposure that may be socioeconomic and cross-cultural in origin. Understanding how individual differences in sensitivity and exposure may arise in diverse participants paves the way for adaptive and personalized interventions, allowing for a more targeted “precision medicine” approach to light exposure.

4. Defining actionable light exposure limits and recommendations.

Global organizations such as the World Health Organization (WHO), as well as transnational, national, and regional public health organizations, must consider clear definitions of exposure limits for light during the biological day and the biological night. Like existing exposure limits for noise, light exposure should be codified. The recently published expert recommendations (Brown et al., 2022) for light levels in the daytime (>250 lx melanopic equivalent daylight illuminance (EDI)), presleep (<10 lx melanopic EDI), and sleep (<1 lx melanopic EDI) can represent a starting point for this, with the caveat that there are time-dependent complexities. There is clear evidence that light exposure during the day can offset some effects of evening/nighttime light exposure (Chang et al., 2011; Hébert et al., 2002; Smith et al., 2004; Te Kulve et al., 2019), indicating the need to develop exposure models that are more complex than the evaluation of instantaneous exposure. Importantly, these limits must include a lifespan view, moving away from the idea of an average observer and incorporating known differences in light sensitivity across the lifespan. Moving beyond prior consensuses (Brown et al., 2022; Moore-Ede et al., 2023), a broad coordinated position statement incorporating the key stakeholders could represent a push toward coordinated changes in policy. In the field of light pollution, recommendations for mitigation with a direct impact on human health have recently been proposed (Zielinska-Dabkowska et al., 2023).

5. Recognizing healthy light exposure as a human right with expected benefits to community health.

Light exposures that support health and wellbeing should be protected by laws and regulations as a fundamental right. This includes both access to sufficient light during the “biological day” and the absence of light during the “biological night.” At one extreme, bright and strobing lights have been misused as torture instruments (Fetherston, 2020), and constant illumination has been used in dwellings for refugee children (Czeisler, 2018). In the community, light exposures are generally known to be suboptimal in terms of timing or intensity for those in workplaces, schools, hospitals, aged care facilities, and prisons. The benefits of improved light exposures are largely speculative at the population level, but optimal exposures would likely improve circadian rhythms, sleep, and their neuroprotective effects. Such effects might be especially powerful for the developmentally vulnerable or chronically ill (Davis et al., 2016; Ricketts et al., 2022; Vásquez-Ruiz et al., 2014). Because electric light is pervasive in the home, workplace, and community, the deployment of more biologically optimal lighting would not necessitate the expensive upgrading of infrastructure. It is therefore a cost-effective method to improve health and wellbeing in the community.

6. Develop evidence-based standards in lighting marketing for nonprofessionals.

The lighting industry is a large market, where LED lighting is valued at >70 billion USD globally and growing yearly. “Human-centric lighting” or “circadian lighting” represents a major recent push of the LED lighting industry toward products that in some way incorporate the recent knowledge on the NIF effects of light on human physiology. These claims can exceed the current state of knowledge (Houser et al., 2021; Joyce et al., 2023), and to the consumer, it may not be obvious to what extent lighting solutions that are available on the market are indeed conducive to health. There are also subtleties of messaging that may not be understood by the consumer (e.g., that blue-enriched light is potentially harmful to circadian rhythms during the evening but potentially helpful during the day). Clear standards must be developed based on scientific evidence that aid consumer decisions in selecting lighting solutions.

## Conclusion

The effects of light are undeniably powerful. New understanding of NIF retinal and brain circuitry heralds the dawn of the melanopsin age where people must reevaluate how both sunlight and electric light are presented in human communities. First and foremost, policies must do no harm. We must then rethink the role of light in society and how to best deploy lighting to maximize human health and wellbeing. In pursuit of this, all of us—lighting researchers, professionals, stakeholders, and community—must work together to enhance our understanding of the effects of light, translate this knowledge to lighting products, and deploy such lighting in responsible and biologically optimal ways. The development and refinement of evidence-based lighting policy is key to driving this agenda.

## Social Media Post

Discover the unseen powers of light! From basic biology to translation, we need to address urgent gaps in the nonvisual effects of light, including personalized, adaptive lighting considering individual differences, and lighting equity.

## Key Points

- The late 1990s revealed, in the retina, the intrinsically photosensitive ganglion cells (ipRGCs), the pathway that signals environmental light levels to the brain.
- These signals underlie the non–image-forming (NIF) effects of light, including synchronizing the circadian clock and modifying hormonal secretions, including melatonin.
- This neurobiological discovery has fundamentally changed the way that the built environment uses light. Electric light has traditionally helped people see well but can now help people to think, act, and feel well.
- A roadmap furthers developing NIF and lighting knowledge, implementing optimal lighting, and recognizing in stakeholder partnerships the fundamental role of light in human health, wellbeing, and behavior.
- Policies need to anchor biologically optimal light as a fundamental human right.
